# Using Bayesian networks with Max-Min Hill-Climbing algorithm to detect factors related to multimorbidity

**DOI:** 10.3389/fcvm.2022.984883

**Published:** 2022-08-30

**Authors:** Wenzhu Song, Hao Gong, Qili Wang, Lijuan Zhang, Lixia Qiu, Xueli Hu, Huimin Han, Yaheng Li, Rongshan Li, Yafeng Li

**Affiliations:** ^1^School of Public Health, Shanxi Medical University, Taiyuan, China; ^2^Department of Biochemistry and Molecular Biology, Basic Medical College, Shanxi Medical University, Taiyuan, China; ^3^The Second Clinical Medical College, Shanxi Medical University, Taiyuan, China; ^4^Department of Nephrology, Shanxi Provincial People’s Hospital (Fifth Hospital) of Shanxi Medical University, Taiyuan, China; ^5^Shanxi Provincial Key Laboratory of Kidney Disease, Taiyuan, China; ^6^Core Laboratory, Shanxi Provincial People’s Hospital (Fifth Hospital) of Shanxi Medical University, Taiyuan, China; ^7^Shanxi Medical University, Academy of Microbial Ecology, Taiyuan, China

**Keywords:** Bayesian networks, multimorbidity, related factors, model construction, Max-Min Hill-Climbing algorithm

## Abstract

**Objectives:**

Multimorbidity (MMD) is a medical condition that is linked with high prevalence and closely related to many adverse health outcomes and expensive medical costs. The present study aimed to construct Bayesian networks (BNs) with Max-Min Hill-Climbing algorithm (MMHC) algorithm to explore the network relationship between MMD and its related factors. We also aimed to compare the performance of BNs with traditional multivariate logistic regression model.

**Methods:**

The data was downloaded from the Online Open Database of CHARLS 2018, a population-based longitudinal survey. In this study, we included 10 variables from data on demographic background, health status and functioning, and lifestyle. Missing value imputation was first performed using Random Forest. Afterward, the variables were included into logistic regression model construction and BNs model construction. The structural learning of BNs was achieved using MMHC algorithm and the parameter learning was conducted using maximum likelihood estimation.

**Results:**

Among 19,752 individuals (9,313 men and 10,439 women) aged 64.73 ± 10.32 years, there are 9,129 ones without MMD (46.2%) and 10,623 ones with MMD (53.8%). Logistic regression model suggests that physical activity, sex, age, sleep duration, nap, smoking, and alcohol consumption are associated with MMD (*P* < 0.05). BNs, by establishing a complicated network relationship, reveals that age, sleep duration, and physical activity have a direct connection with MMD. It also shows that education levels are indirectly connected to MMD through sleep duration and residence is indirectly linked to MMD through sleep duration.

**Conclusion:**

BNs could graphically reveal the complex network relationship between MMD and its related factors, outperforming traditional logistic regression model. Besides, BNs allows for risk reasoning for MMD through Bayesian reasoning, which is more consistent with clinical practice and thus holds some application prospects.

## Introduction

Multi-morbidity (MMD) is defined as the co-occurrence of two or more long-term health conditions in the same individual (1). According to a previous study, the prevalence was projected at 49.64% in the elderly in China (2). Due to its high morbidity, high prevalence, high mortality, insidious, and non-specific characteristics, MMD patients have often missed the best treatment time window, seriously affecting the quality of life of the elderly (3). Additionally, in rural areas, the backward medical resources, coupled with lower awareness of physical examination, are responsible for a higher incidence of MMD.

Amid an aging society with an increasing medical burden and rising demand for medical treatment, improving our understanding of MMD and realizing their comprehensive management, improving the maintenance of the health of the whole people, standardizing living habits, and avoiding the misunderstanding of MMD has become the key to solving the primary public health problems currently facing the international community, and are also the core part of China’s realization of the goal of “Healthy China 2030.” Although the 13th 5-Year Plan in China has incorporated chronic disease management into the national priority strategy, the treatment of MMD is still in its infancy compared with developed countries because the treatment of MMD is currently too simple. Early identification and prevention of MMD-inducing factors still represent an effective way to reduce the prevalence of MMD. Therefore, analysis of relevant related factors and risk reasoning of MMD could provide new ideas for clinicians to diagnose, and treat MMD early, thus prevention and control strategies could be taken accordingly.

It has been documented that logistic regression model was employed to discuss the factors related to MMD with both cross-sectional data (4) and longitudinal data (5), suggesting that age, sex, smoking, drinking, sleep duration, physical activity, etc. are associated with MMD occurrence. Yet, the model is accompanied by some defects. The first relates to independent variable. In clinical studies, factors tend to be correlated, and the model fails to meet the prerequisite of independence between variables. The second one lies in its inability to make sequential predictions. Third, the model could not explore the network relationship between related factors and disease, unable to identify direct or indirect related factors (6).

In recent years, Bayesian Networks (BNs) (7) pick up pace in medical research. It comprises a directed acyclic graph (DAG) and a conditional probability distributions table (CPT), the former of which allows for potential links between one specific disease and its related factors, and between one related factor and other related factors. The latter could reflect the associations between variables, facilitating an explanation of the complex internal relationships between diseases and their related factors. Without strict statistical assumptions (8), BNs represent a good approach for sequential risk predictions. The learning of BNs includes structural learning and parameter learning. Structural learning could be achieved by Constraint-based (CB) algorithms and Scoring and searching (SS) based algorithms. The former features high learning efficiency, allowing for the global optimal solution, but it’s subject to complicated conditional independence judgment and not necessarily reliable independence results in high-level conditional tests. The latter could search for a more accurate network structure, yet determining the optimal one from all possible structures was proven an NP-hard problem. Max-Min Hill-Climbing (MMHC) algorithm is a hybrid algorithm (6) that could allow for the combination of both SS algorithm and CB algorithm, thereby, offering higher accuracy.

In this paper, we aimed to construct an MMHC algorithm-based BNs, using Online Open Database, CHARLS 2018, to explore the factors associated with MMD; we also aimed to investigate its performance with traditional logistic regression model, discussing the application prospects of BNs in clinical practice, and thus offering a feasible suggestion for improving older people’s quality of life.

## Materials and methods

### Study participants

This data is downloaded from The China Health and Retirement Longitudinal Study (CHARLS) 2018,^1^ which was released on September 23. It’s a longitudinal survey of residents aged Chinese mainland 45 and above (9, 10), covering 150 districts and 450 villages/urban communities across the country, involving 19,752 people in 10,257 families, comprehensively reflecting the collective situation of China’s middle-aged and elderly population. Informed consent was signed by all respondents and all CHARLS waves are ethically approved by the Institutional Review Committee of Peking University.

Using a multi-stage stratified group random sampling (11), 9 provinces were randomly selected in the eastern, central and western regions of China, and then the counties of each province were stratified following the income scale (low, medium and high). Finally, a total of 36 counties and 108 villages, and about 220 community samples were sampled. A questionnaire survey was used to collect demographic information (sex, age, education levels, marital status, residence) and lifestyle (smoking, alcohol consumption, physical activity, nap, sleep duration) of the population participating in the survey in 2018. Some of the data was partly missing and we used Random Forest to handle the problem. Of note, CHARLS also made a survey on Family Transfer, Family Information, Work Retirement, Pension, and Household Income, which may also serve as potential factors related to MMD. Yet, previous studies primarily focused on demographic information and lifestyle-related factors. As far as we know, few researchers employ BNs to detect the complex network relationship between these factors. As such, we chose these variables as our study variables.

### Variable definition

The age classification is <55 years old, 55–65 years old, 65–75 years old, and ≥75 years old. Marital status is divided into Married, Divorced, Widowed, and Never Married. The education levels are divided into incomplete primary school (≤primary school), primary school/junior high school (≤high school), high school/secondary school/junior college (<college), undergraduate, and above (≥college). The residence is divided into Town, Combination zone between urban and rural areas (boundary), Village, Special area. Smoking is defined as No and Yes. Alcohol consumption is defined as No or Yes. The nap duration is divided into 0 min, 0∼30 min ≥ 30 min. Sleep duration is divided into ≤5 h, 5–6 h, 6–7 h, 7–8 h, and ≥ 8 h.

The International Physical Activity Questionnaire (IPAQ) was used to obtain the physical activity of the study subjects and calculate the physical activity energy expenditure: the corresponding exercise intensity assignment for this physical activity × the weekly frequency (d/w) × the time per day (min/d), and the energy expenditure of the three intensities is the total physical activity consumption of 1 week. The MET assignment for vigorous-intensity physical activity is 8.0, the moderate-intensity assignment is 4.0, and the light-intensity assignment is 3.3. Based on the calculated physical activity energy expenditure, physical activity is divided into three mutually exclusive groups: Light (<600 MET-min/week), Moderate (600–3,000 MET-min/week), and Vigorous (≥3,000 MET-min/week) (12, 13).

The CHARLS database collected self-reported medical information based on the doctor’s diagnosis, and CHARLS asks a total of 14 types of chronic diseases diagnosed by the doctor which was achieved by asking “Have you been diagnosed with the following conditions by a doctor,” including hypertension, dyslipidemia, diabetes, cancer, chronic lung disease, liver disease, heart disease, stroke, kidney disease, stomach or digestive system disease, emotional or psychiatric problems, memory-related diseases (such as Alzheimer’s disease, brain atrophy, Parkinson’s), arthritis or rheumatism, asthma. All diseases or conditions are defined as binary variables (presence and absence). Multi-morbidity is defined as a person suffering from two or more chronic diseases/conditions in an individual (14).

### Bayesian networks

BNs are made up of a DAG and a CPT (4). The former consists of nodes and edges; nodes mean variables in the network and if variable A points to variable B, it suggests a direct probability dependency between A and B. We also name A as the parental node of B and B as the child node of B. CPT quantitatively describes the degree of probability dependence of a node and its parent node (5). Thus, BNs use the graphical structure and network parameters to uniquely determine the joint probability distribution on the random variable = *x*{X1, X*n*}, which can be listed as:


(1) $$\text{P}\left(x_{1},x_{2},\ldots ,x_{n}\right)=\text{P}\left(x_{1}\right)\text{P}\left(x_{2}|x_{1}\right)\cdots \text{P}\left(x_{n}|x_{1},x_{2},\ldots ,x_{n-1}\right)$$

$$=\Pi_{1}^{\text{n}}\text{P}\left(x_{i}|\pi \left(x_{i}\right)\right)$$

π(*x*_*i*_) is the set of parent nodes of *x*_i_,π(*x*_i_)⊆{*x*_1_,*x*_2_,…,*x*_*i*−1_}. When the value of π(*x*_*i*_) is known, *x_i_* is conditionally independent of other variables in {*x*_1_,*x*_2_,…,*x*_*i*−1_}.

### Max-Min Hill-Climbing algorithm

Structural learning of BNs is primarily implemented by Constraint-based (CB) algorithms and Scoring and searching (SS) based algorithms. Constraint-based algorithms use conditional independence tests to learn conditional independence constraints from data. The constraints in turn are used to learn the structure of the BN under the assumption that conditional independence implies graphical separation (so, two independent variables cannot be connected by an arc). Score-based algorithms are general-purpose optimization algorithms that rank network structures concerning a goodness-of-fit score. MMHC algorithm represents a hybrid algorithm that combines both constraint-based and score-based algorithms (6), as they use conditional independence tests (usually to reduce the search space and network scores to find the optimal network in the reduced space) simultaneously (7).

### Statistical analysis

Qualitative data were expressed as percentages (%). The variables are included into both logistic regression model construction and the BNs construction. The result of logistic regression model was visualized using the forest plot. The structure learning of BNs is achieved using the MMHC() function in the package “bnlearn” in R software. The parameter learning of BNs is carried out using the maximum likelihood estimation. Last, the BNs and conditional probability distribution table are plotted by Netica software.

## Results

### Baseline characteristics of respondents

A total of 19,752 individuals were enrolled in the study, including 10, 623 patients with MMD, of whom 4,806 were men (45.2%) and 5,817 were women (54.8%). Among the ages < 55 years, 55–65 years old, 65–75 years old and > 75 years old, the proportion was 13.8, 31.4, 35.0, and 19.8%, respectively. The proportions of physical activity in light activity, moderate activity and heavy activity accounted for 3.5, 31.0, and 65.4%, respectively. The proportion of ≤ primary school, ≤ middle school, < college, ≥ college represented 41, 44.9, 13.1, and 0.9%, respectively. Among the individuals without MMD, 4,507 were men (49.4%) and 4,622 (50.6%) were women. Among the ages < 55 years, 55–65 years old, 65–75 years old, and > 75 years old, the proportions occupied 22.1, 35.1, 27.5, and 15.3%. The proportions of physical activity in light activity, moderate activity and heavy activity constituted 4.3, 33.9, and 61.8%, respectively. The proportion of ≤ primary school, ≤ middle school, < college, ≥ college represented was 45.6, 42.9, 10.8, and 0.8%, respectively. Other detailed information could be available in Table 1.

**TABLE 1:** Baseline characteristics of individuals with and without multimorbidity.

Variables	Without multimorbidity (*n* = 9,129)	With multimorbidity (*n* = 10,623)
**Sex**		
Male	4,507 (49.4)	4,806 (45.2)
Female	4,622 (50.6)	5,817 (54.8)
**Age**		
<55	2,018 (22.1)	1,466 (13.8)
55∼65	3,202 (35.1)	3,334 (31.4)
65∼75	2,514 (27.5)	3,719 (35.0)
≥75	1,395 (15.3)	2,104 (19.8)
**Physical activity**		
Light	324 (3.5)	454 (4.3)
Moderate	2,832 (31.0)	3,605 (33.9)
Vigorous	5,973 (65.4)	6,564 (61.8)
**Education levels**		
≤Primary school	3,744 (41)	4,844 (45.6)
≤Middle school	4,101 (44.9)	4,554 (42.9)
<College	1,200 (13.1)	1,142 (10.8)
≥College	84 (0.9)	83 (0.8)
**Residence**		
Town	1,709 (18.7)	1,892 (17.8)
Combination	640 (7.0)	788 (7.4)
Village	6,738 (73.8)	7,898 (74.3)
Special area	42 (0.5)	45 (0.4)
**Marital status**		
Married	7,977 (87.4)	8,912 (83.9)
Divorced	111 (1.2)	130 (1.2)
Widowed	982 (10.8)	1,522 (14.3)
Never married	59 (0.6)	59 (0.6)
**Sleep duration**		
≤5	2,408 (26.4)	3,951 (37.2)
5∼6	2,082 (22.8)	2,235 (21)
6∼7	1,754 (19.2)	1,630 (15.3)
7∼8	1,957 (21.4)	1,860 (17.5)
≥8	928 (10.2)	947 (8.9)
**Nap**		
0	3,589 (39.3)	3,992 (37.6)
0∼30	1,573 (17.2)	1,910 (18.0)
≥30	3,967 (43.5)	4,721 (44.4)
**Smoking**		
No	5,315 (58.2)	6,215 (58.5)
Yes	3,814 (41.8)	4,408 (41.5)
**Alcohol consumption**		
No	5,800 (63.5)	7,310 (68.8)
Yes	3,329 (36.5)	3,313 (31.2)

### Results of logistic regression

**FIGURE 1:**
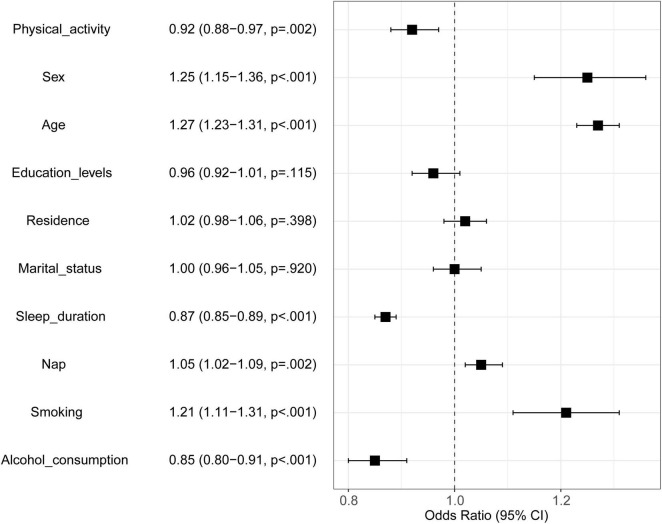
Result of traditional logistic regression model. Black square represents Odds Ratio; the two ends of the square represent the 95% confidence interval (95% CI). If 95% CI crosses through the dotted line, it indicates that the corresponding variable is not correlated with MMD.

In this study, the 10 variables were included into logistic regression model construction for factors associated with MMD. Variables and their assignments were shown in Table 2. The forest plot was used to visualize the result of logistic regression. As demonstrated in Figure 1, physical activity (OR = 0.92, 95%CI: 0.88-0.97), sex (OR = 1.25, 95%CI: 1.15-1.36), age (OR = 1.27, 95%CI: 1.23-1.31), sleep duration (OR = 0.87, 95%CI: 0.85-0.89), nap (OR = 1.05, 95%CI: 1.02-1.09), smoking (OR = 1.21, 95%CI: 1.11-1.31) and alcohol consumption (OR = 0.85, 95%CI: 0.80-0.91) are associated with MMD (*P* < 0.05). Yet, education levels, residence, and marital status were not associated with MMD (*P* > 0.05).

**TABLE 2:** Variables and their assignments.

Variables	Assignments
Physical activity (x_1_)	Light = 1; moderate = 2; vigorous = 3
Sex (x_2_)	Men = 1; women = 2
Age (x_3_)	≤ 55 = 1; ≤65 = 2; ≤75 = 3; > 75 = 4
Education levels (x_4_)	≤ Primary = 1; ≤high = 2; ≤college = 3; > college = 4
Residence (x_5_)	Urban = 1; boundary = 2; rural = 3; special = 4
Marital status (x_6_)	Married = 1; divorced = 2; widowed = 3; never married = 4
Sleep duration (x_7_)	≤ 5 h = 1; ≤6 h = 2; ≤7 h = 3; ≤8 h = 4; > 8 h = 5
Nap (x_8_)	0 = 1; ≤ 30 min = 2; > 30 min = 3
Smoking (x_9_)	No = 0; yes = 1
Alcohol consumption (x_10_)	No = 0; yes = 1
MMD (y)	No = 0; yes = 1

### Bayesian networks

**FIGURE 2:**
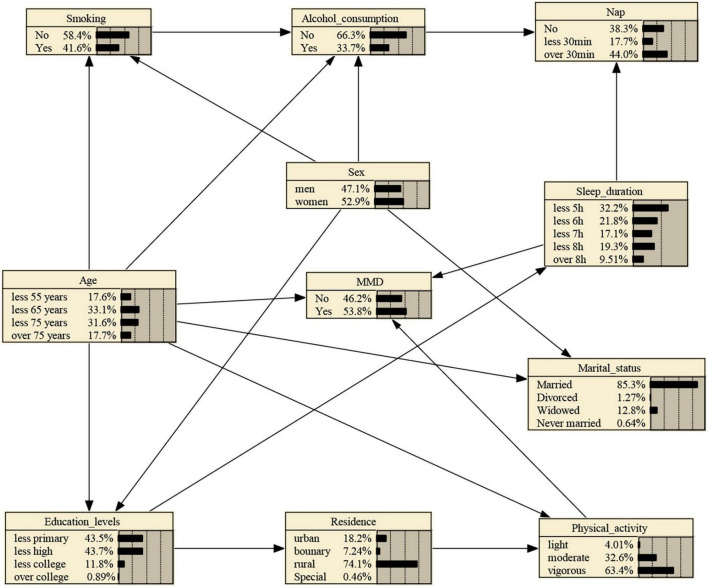
MMD Bayesian networks and prior probability using MMHC Algorithm. The networks were constructed with 11 nodes and 18 directed edges. Node represents variable, and directed edges represent probabilistic dependence between connected nodes. The percentage in the figure means the prior probability of each node. Boundary: Combination zone between urban and rural areas.

Likewise, the 10 variables were included into BNs construction. In this study, BNs were constructed with 11 nodes and 18 directed edges. Node represents variable, and directed edges represent probabilistic dependence between connected nodes. The percentage in the figure means the prior probability of each node. As shown in Figure 2, the prior probability of MMD represents 0.538, i.e., P (MMD) = 0.538. BNs showed that age, sleep duration, and physical activity are the parental nodes of MMD, suggesting that age, sleep duration, and physical activity are directly linked to MMD. Additionally, sex, nap, smoking, and alcohol consumption are indirectly associated with MMD. Also, age is the parental node of physical activity, which means age could directly influence MMD and could indirectly influence MMD by physical activity. Besides, education levels are directly related to residence, which could indirectly influence MMD through sleep duration, showing the BNs could reveal intermediate links between related factors and disease occurrence. Besides, we could learn that sex and age are the parental nodes of both education levels and marital status, suggesting sex and age have a direct correlation with education levels and marital status.

### Bayesian reasonings

BNs could infer unknown nodes from known nodes, enabling risk prediction of disease occurrence. That is to say, the probabilistic model could quantitatively analyze the influence of these factors on MMD *via* computing conditional probabilities P (y| xi).

If one is subject to light physical activity, the probability increases from the prior probability to 0.579, that is, P (MMD| light physical activity) = 0.579, as shown in Supplementary Figure 1. And if the person’s sleep duration stands at ≤ 5 h, the probability rises to 0.620, that is, P (MMD| light physical activity, ≤ 5 h sleep duration) = 0.620, as shown in Supplementary Figure 2. Besides, If the person’s age is between 65 and 75 years, the probability rises to 0.671, that is, P (MMD| light physical activity, ≤ 5 h sleep duration, ≤ 75 years) = 0.671, as shown in Supplementary Figure 3.

## Discussion

In this paper, the BNs with the MMHC algorithm was used to explore the related factors of MMD, which not only shows the direct and indirect factors for MMD but also realizes the risk reasoning of MMD. To the best of our knowledge, little attention has been poured into BNs with MMHC algorithm to discuss the factors associated with MMD. The results showed age, sleep duration, and physical activity are directly related to MMD. Scholars usually employ logistic regression model, with probabilities reflecting the strength of the association, to detect the related factors for MMD, suggesting that sex, age, smoking, and alcohol consumption etc. represented risk factors for MMD (14). Yet the model comes with some disadvantages. The first one is that the model fails to explore the direct or indirect factors associated with MMD. The second one lies in its inability to make a sequential prediction (8).

BNs outperform logistic regression. On the one hand, logistic regression often fits the regression model under the assumption that the variables are independent of each other, failing to make full use of data information (6), and unable to reflect the impact of the risk factors on MMD and the relationship. Additionally, the model has no strict requirements for the distribution of data, so it can fully explore the potential information of the data, reveal the interrelationship between factors, and provide a scientific basis for the evaluation, prediction and prevention of MMD. On the other hand, logistic regression analysis can only reveal several independent influencing factors of MMD, while BNs allow for further description of how the related factors are interrelated and affect the occurrence of MMD through a graphical approach (15). In this study, education levels, residence, and marital status were not shown to be associated with MMD using logistic regression model. Yet, BNs showed that education levels could be directly connected to residence, which then could be indirectly associated with MMD through physical activity, suggesting its capability to detect the intermediate links between related factors and MMD, and its suitability for searching new variables associated with MMD. As such, BNs could detect the role of various related factors in the occurrence of MMD.

With aging population, improved living standards, increased societal pressure and changing daily lifestyles, MMD has not only emerged as a major public health issue but has also become a serious economic problem, putting a damper on social development. It could be justified to strengthen the formulation, monitoring and evaluation of the national chronic disease prevention and control plan, and improve people’s understanding of factors related to MMD.

An increasing age comes with lower immunity, less adaptability to the external environment, and a heavier emotional backlog burden, leading to a higher probability of MMD occurrence. Besides, Aging, melatonin deficiency will gradually cause sleep cycle disorders (16, 17). Meanwhile, the hippocampus occurs lipid peroxidation, resulting in memory loss in the elderly, and emotional interest loss, and further increases the risk of MMD. Aortic stiffness increases with age, aging aortic intima thickens, total collagen content increases, and elasticity decreases, resulting in hardening of the arteries. The American Heart Association (AHA) suggests that aortic stiffness is a major cause of high blood pressure in the elderly (18). Our study demonstrated that sleep deprivation is highly correlated with the incidence of multiple chronic diseases, which is consistent with the previous studies (19, 20).

Ucar et al. suggest that (21) sleep duration <6 h is closely related to coronary heart disease and obstructive sleep apnea syndrome. Lack of sleep can affect circadian rhythms, reduce insulin sensitivity, increase insulin resistance, and cause catecholamine and cortisol levels to rise, increasing the incidence of type 2 diabetes. Sleep disturbances can cause increased sympathetic excitability, increase resting heart rate, increase myocardial oxygen consumption; Constriction of peripheral blood vessels, and increased peripheral blood pressure, causing essential hypertension; Sympathetic activity is one of the main factors of ventricular remodeling, α adrenaline receptors, β adrenaline receptors and norepinephrine are closely related to the occurrence and development of myocardial hypertrophy and fibrosis, which indicates that sleep disorders are associated with the development of hypertension (22).

Sedentary increases the production of reactive oxygen species, leading to a chronic state of oxidative stress, in type II diabetes, oxidative stress changes the secretion of insulin and the sensitivity of hormones to target cells, so the lack of exercise increases the prevalence of diabetes (23). Aerobic exercise can promote metabolism, and improve the function of islet β cells to regulate blood sugar (24). Exercise can improve vascular function and myocardial remodeling, prevent depression, reduce hypoxia, and promote blood circulation (25). Meanwhile, exercise can also adjust the volume of the chest cage, optimize the breathing mode of patients with chronic obstructive pulmonary disease, and help cough up sputum and calm asthma.

This study should be interpreted in the context of several limitations. First, it’s a cross-sectional study and BNs is a data-driven model, it could not reflect the causal but correlated relationship between MMD and its related factors. Dynamic BNs and multilevel temporal BNs could be employed to fully explore the deeper relationship between MMD and its related factors. Second, since the data was obtained using a self-report questionnaire, it may underestimate the prevalence of MMD, especially in older people and those with lower socioeconomic and educational backgrounds. Besides, in addition to the variables included in this study, more other variables should be included to comprehensively explore the factors associated with MMD. Also, we did not conduct hierarchical regression model completed with mediation analysis to make an analysis, which will also be the focus of our future work. Last, the target outcome is defined as having 2 or more diagnoses of chronic conditions, which may be insufficiently reliable from the practical perspective. Our next work should discuss the most common disease combination as the target outcome variable.

In conclusion, BNs has advantages over logistic regression in exploring related factors for MMD, allowing for graphical demonstration of complex network relationship between MMD and its related factors. Besides, Bayesian reasoning makes risk prediction for MMD possible, which could offer help in clinical practice and have application prospects.

## Data availability statement

The raw data supporting the conclusions of this article will be made available by the authors, without undue reservation.

## Ethics statement

Informed consent was signed by all respondents and all CHARLS waves are ethically approved by the Institutional Review Committee of Peking University.

## Author contributions

WS drafted the manuscript. HG, QW, and LZ helped make a data analysis and polish the manuscript. LQ, XH, HH, YhL, and RL gave precious advice on the statistical methods. YfL was responsible for the conception and design of the research. All authors contributed to the article and approved the submitted version.

## Footnotes

1
http://charls.pku.edu.cn/en/

## Supplementary material

Supplementary Figure 1 Bayesian reasoning for MMD under light physical activity.

Supplementary Figure 2 Bayesian reasoning for MMD under light physical activity and sleeping duration less than 5 hours.

Supplementary Figure 3 Bayesian reasoning for MMD under light physical activity, sleeping duration less than 5 hours, and with an age of 65–75 years.
