# Supplementary figures and images for: Using Bayesian networks with Max-Min Hill-Climbing algorithm to detect factors related to multimorbidity
Source: Front Cardiovasc Med. 2022 Aug 30;9:984883. doi: 10.3389/fcvm.2022.984883 (PMC9468216; doi:10.3389/fcvm.2022.984883)

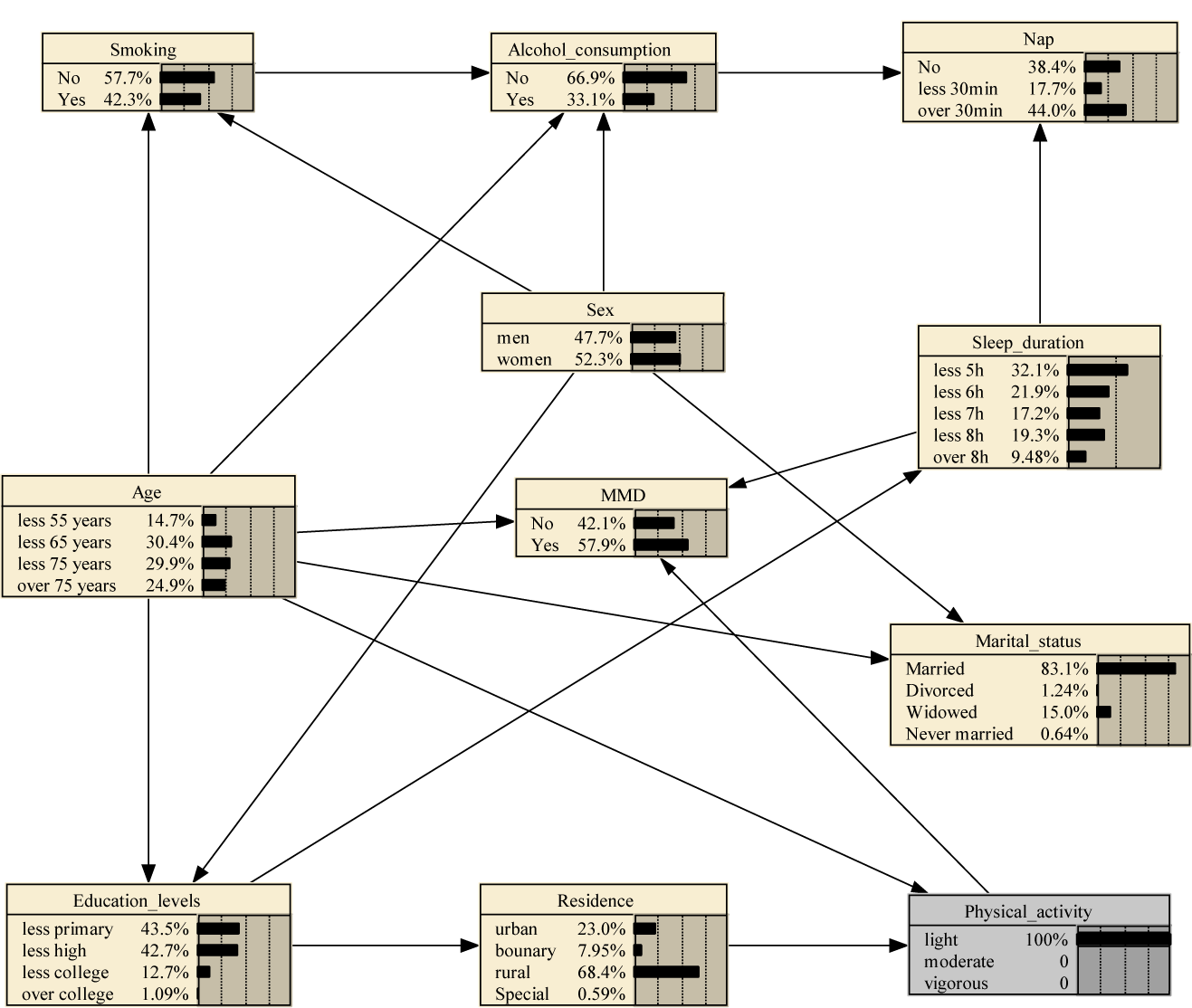

Supplement: Supplementary Figure 1 — Bayesian reasoning for MMD under light physical activity. [file Image_1.tif]

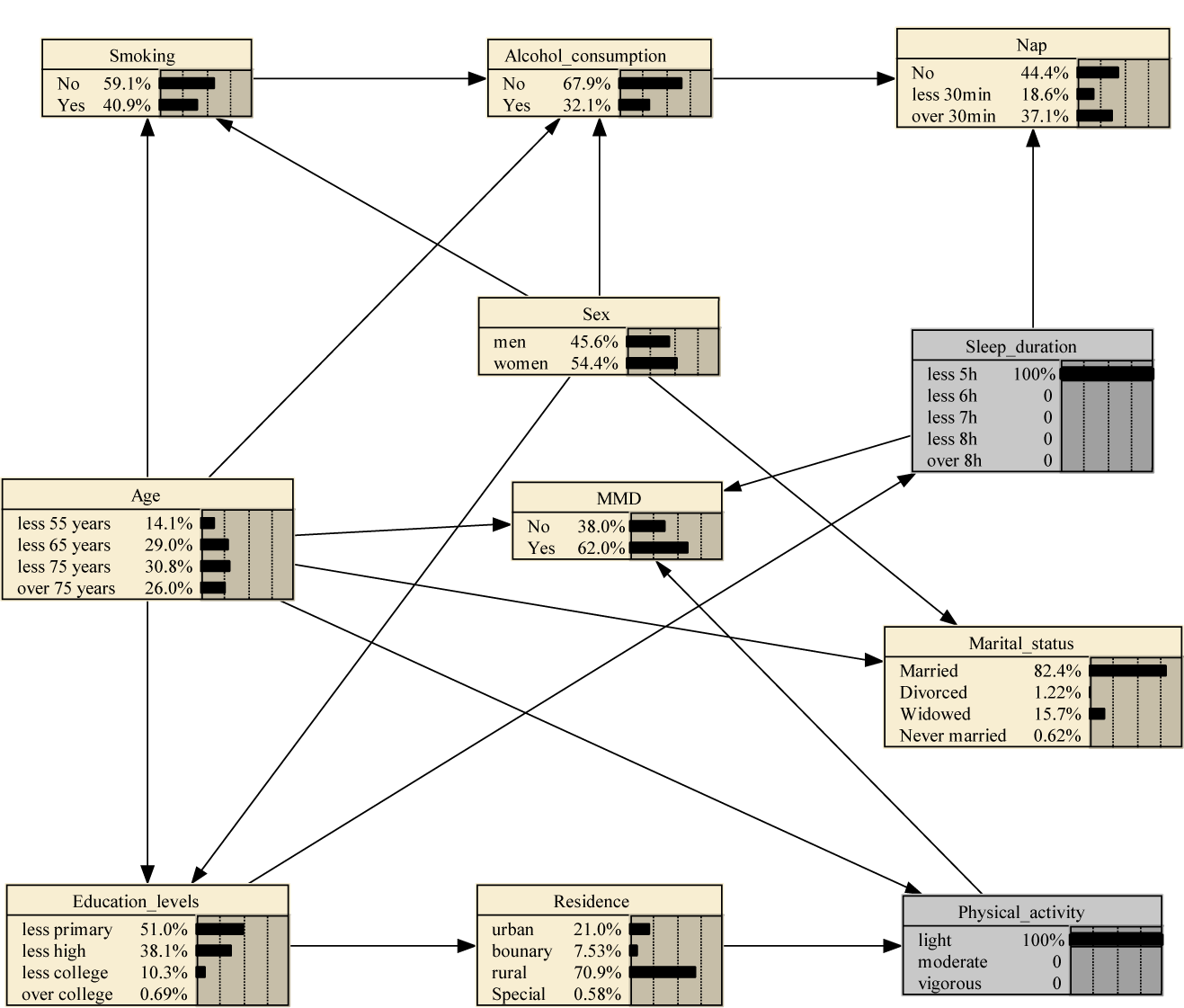

Supplement: Supplementary Figure 2 — Bayesian reasoning for MMD under light physical activity and sleeping duration less than 5 hours. [file Image_2.tif]

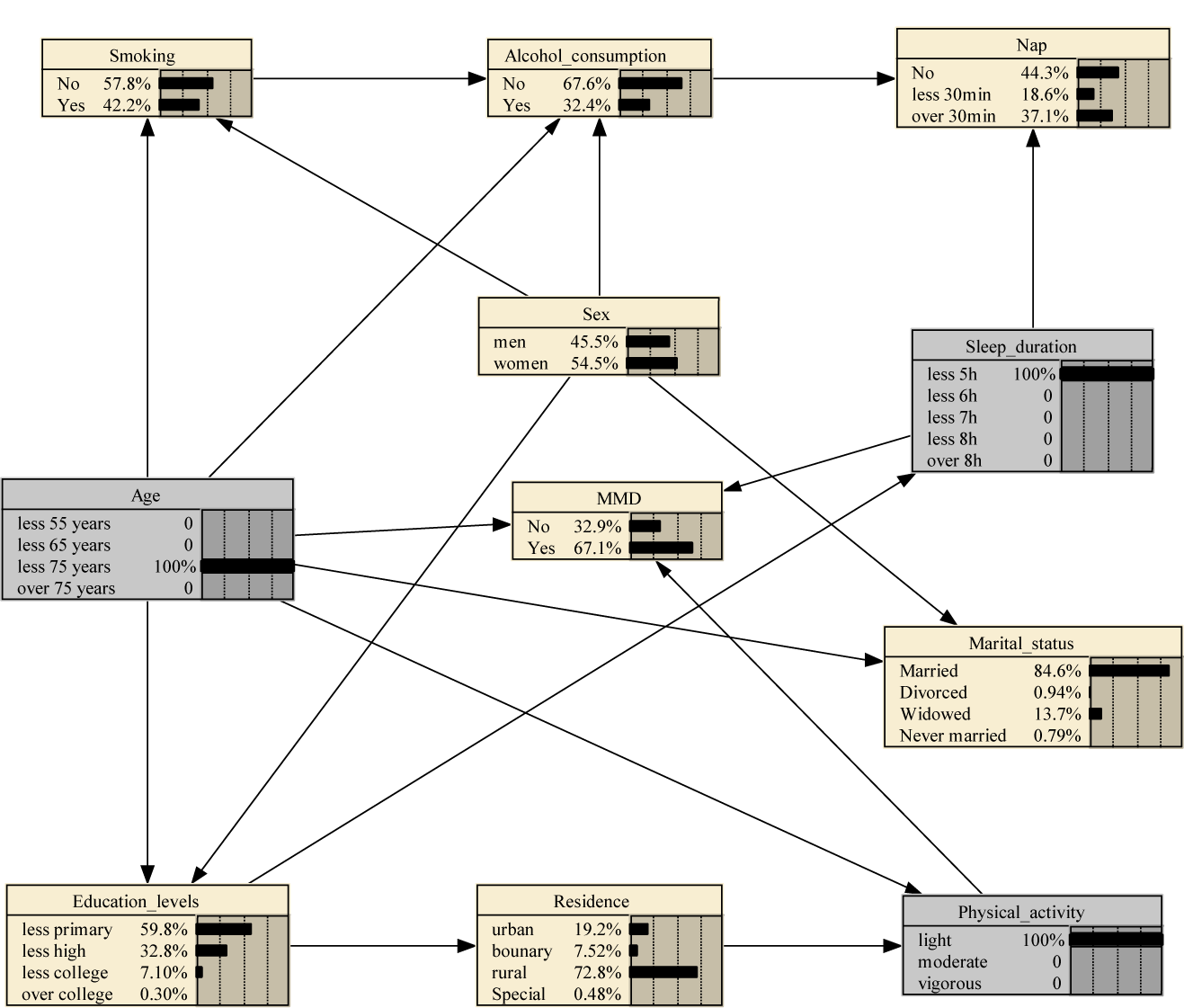

Supplement: Supplementary Figure 3 — Bayesian reasoning for MMD under light physical activity, sleeping duration less than 5 hours, and with an age of 65–75 years. [file Image_3.tif]
